# A Rare Case of Cardiac Tamponade Induced by Chronic Lymphocytic Leukemia

**DOI:** 10.7759/cureus.51271

**Published:** 2023-12-29

**Authors:** Catherine G Raciti, Hazem Alakhras, Kateryna Strubchevska, Steven Timmis, Marko Kozyk

**Affiliations:** 1 Internal Medicine, Oakland University William Beaumont School of Medicine, Royal Oak, USA; 2 Internal Medicine, Corewell Health William Beaumont University Hospital, Royal Oak, USA; 3 Cardiovascular Medicine, Corewell Health William Beaumont University Hospital, Royal Oak, USA

**Keywords:** chronic lymphocytic leukemia/small lymphocytic lymphoma, pericardiocentesis, cardiac tamponade, pericardial effusion, pericardial window, transthoracic echocardiography (tte)

## Abstract

While cardiac tamponade is a commonly recognized complication in solid organ malignancies and acute leukemias, instances of cardiac involvement in the context of chronic hematologic malignancies, such as chronic lymphocytic leukemia/small lymphocytic lymphoma (CLL/SLL), are rarely observed. A 66-year-old male, with a history of stage IV CLL/SLL, presented with three weeks of worsening edema, orthopnea, and dyspnea. Two days after admission, an echocardiogram revealed a large circumferential pericardial effusion. Given the concern about early signs of pericardial tamponade, the patient underwent emergent pericardiocentesis with the removal of 700 cc of sanguineous fluid. A pericardial biopsy and flow cytometry of the pericardial fluid confirmed the diagnosis of CLL/SLL with pericardial involvement. There were no signs of large cell lymphoma transformation at that point. This rare case demonstrates the importance of considering cardiac complications in CLL/SLL patients who present with worsening edema, orthopnea, and dyspnea.

## Introduction

Many conditions cause pericardial effusion, such as inflammatory disorders, renal disease, trauma, autoimmune conditions, and malignancy [1]. Currently, malignancy accounts for 26% of pericardial effusions in the United States, but only 4.8% of those are due to hematologic malignancy [1].

In patients over 65 years, chronic lymphocytic leukemia/small lymphocytic lymphoma (CLL/SLL) is the most common leukemia in the United States [2]. However, most cases of CLL/SLL have an indolent clinical course, and cardiac involvement is extremely rare [3]. Pericardial effusion with tamponade physiology is an oncological emergency and an unusual manifestation of disease progression in the context of CLL/SLL. However, it is an extremely important diagnosis as it is life-threatening and an indication to start treatment, which can have a significant impact on overall survival [4]. Here, we present a unique case of a 66-year-old male who was diagnosed three years ago with CLL/SLL and presents with progressively worsening edema, dyspnea, and orthopnea. This is an uncommon case of cardiac tamponade secondary to CLL/SLL given that the literature search only showed 18 reported cases of cardiac involvement secondary to this malignancy [3].

## Case presentation

A 66-year-old male, with a history of CLL/SLL, presented with three weeks of progressive exertional dyspnea, orthopnea, and lower extremity edema. He reported associated fatigue but denied fever, weight loss, chest pain, palpitations, and wheezing. Three years prior, he was diagnosed with stage Rai IV CLL/SLL. Throughout the three years since his initial diagnosis, the patient has been on active surveillance, not requiring any interventions up to this point, including chemotherapy and immunotherapy. The only medication he was taking at the time of presentation was Keppra, indicated for his history of seizures.

On initial examination, he was hemodynamically stable and afebrile, with a heart rate of 120 beats per minute, respiratory rate of 18 breaths per minute, and blood pressure of 111/81 mmHg. He appeared non-toxic and was resting comfortably in bed. On examination of the head, eyes, ears, nose, and throat, multiple cervical and supraclavicular non-tender lymphadenopathy were noted. The cardiac assessment revealed regular S1 and S2 heart sounds, accompanied by tachycardia, without murmurs. A respiratory exam revealed clear lung sounds bilaterally in the upper lung lobes, without wheezes or rales. However, fine crackles were heard bilaterally in the lower lung fields, but the patient exhibited normal respiratory effort. In the genitourinary and extremity examination, there was no suprapubic or costovertebral angle tenderness, but scrotal edema was observed along with +3 pitting edema in both lower extremities.

**Figure 1:**
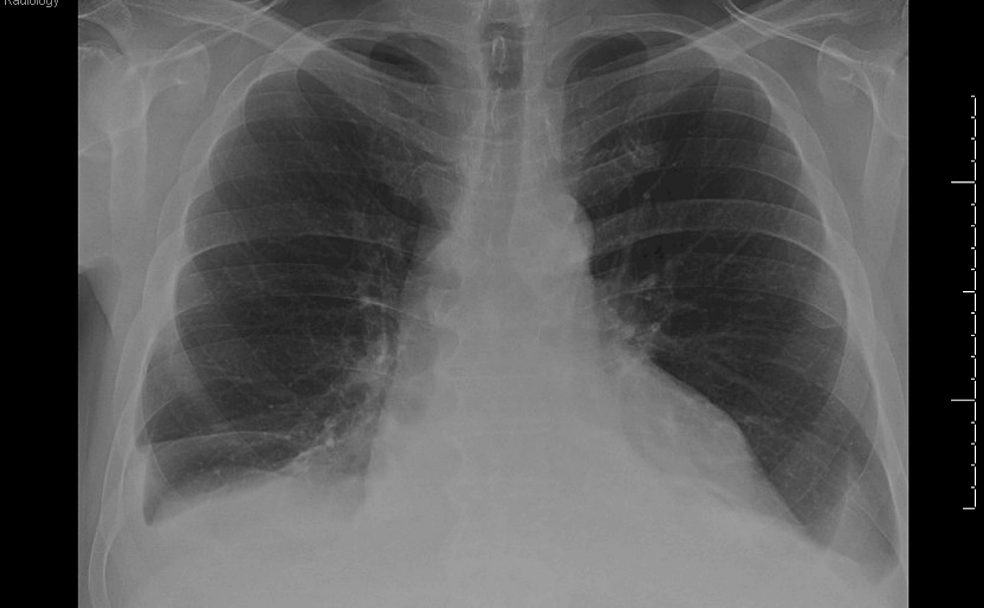
Chest x-ray depicting a small bilateral pleural effusion.

**Figure 2:**
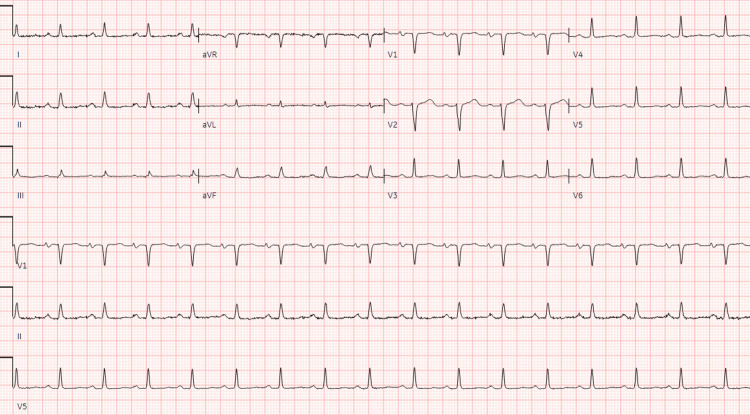
EKG showing low-voltage QRS complexes.

**Figure 3:**
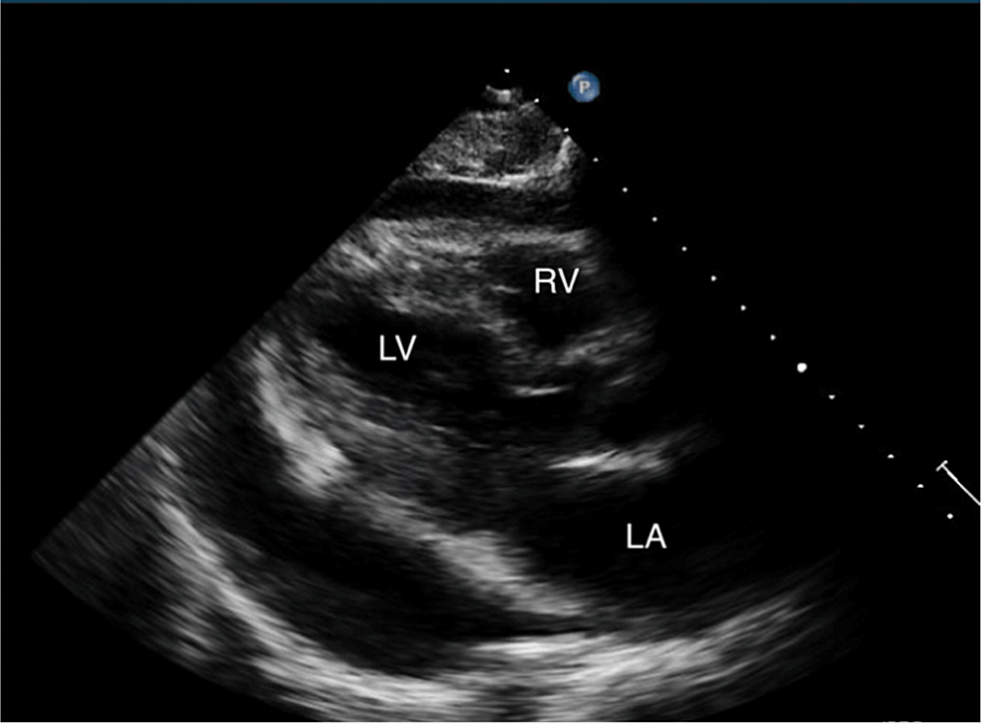
Transthoracic echocardiogram depicting a large pericardial effusion.

The initial electrocardiogram (EKG) showed sinus tachycardia without peaked T-waves or QRS changes. The initial QT interval was 304 ms with a QTc interval of 401 ms. Complete blood count (CBC) demonstrated a decrease in hemoglobin from his baseline of 12.1 g/dL to 10.8 g/dL, along with evidence of leukocytosis, indicated by a count of 178.9 bil/L. Lactate dehydrogenase (LDH) was elevated at 282. A comprehensive metabolic panel (CMP) revealed an elevated potassium of 7.3 mmol/L and a reduced sodium level of 121 mmol/L. More comprehensive laboratory results are shown in Table 1. He was given 1L bolus in the emergency department, followed by IV furosemide 20 mg, as well as IV calcium gluconate, albuterol nebulization, IV insulin, and IV dextrose for the treatment of hyperkalemia. Chest x-ray (CXR) was negative for pulmonary edema or cardiomegaly, but there were small bilateral pleural effusions (Figure 1). In the emergency department, the patient received two more doses of IV furosemide 40 mg and showed no clinical improvement. Furthermore, the patient did not produce any significant amount of urine, although he had not been on any diuretics before. Two days later, a repeat EKG demonstrated sinus tachycardia with low voltage in the limb leads, which was a new finding from the initial EKG (Figure 2). Furthermore, the QT interval increased from the initial EKG to 340 ms with a QTc interval of 460 ms. On follow-up examination, he had a heart rate of 101 beats per minute, respiratory rate of 15 breaths per minute, and a blood pressure of 105/62 mmHg with a pulsus paradoxus of 20 mm Hg. A transthoracic echocardiography (TTE) was conducted and demonstrated an ejection fraction of 60%. Furthermore, it revealed a large circumferential pericardial effusion, with a respiratory variation of mitral and tricuspid inflow, as well as right atrial diastolic collapse (Figure 3). The inferior vena cava was dialated with no respiratory variation indicating an elevated right atrial pressure.

**Table 1:** Laboratory values. BUN: blood urea nitrogen; AST: aspartate aminotransferase; ALT: alanine aminotransferase; WBC: white blood cell; LDH: lactate dehydrogenase; BNP: brain natriuretic peptide

	Day of Presentation	Reference Ranges
Creatinine (mg/dL)	0.86	Adult male: 0.6-1.2
BUN (mg/dL)	27	6-20
Potassium (mmol/L)	7.3	3.5-5.0
Sodium (mmol/L)	121	135-145
Phosphorus (mg/dL)	3.8	2.5-4.5
Calcium (mg/dL)	8.5	8.5-10.5
AST (IU/L)	17	10-40
ALT (IU/L)	18	7-56
WBC (bil/L)	178.9	4-11
Hemoglobin (g/dL)	10.8	Adult male: 13.8-17.2
Platelet (bil/L)	204	150-450
LDH (U/L)	282	140-280
BNP (pg/dL)	72	< 100

Given the concern about early signs of pericardial tamponade, the patient underwent emergent fluoroscopy and ultrasound-guided pericardiocentesis with the removal of 700 cc of bloody fluid, which tested positive for circulating monotypic B-cell population with CLL/SLL phenotype. There were 15,920/mcL of total nucleated cells with 29% neutrophils, 61% lymphocytes, 8% monocytes, and 2% macrophages. Additionally, 91% of the total cells within the pericardial fluid were monotypical B-cells. These B-cells expressed several antigens, including CD5, CD19, CD20, CD22, CD23, CD45, CD200 (bright), and dim kappa light chain. However, they were negative for CD10, CD38, and CD79b. T-cells, accounting for 2% of total cells, had a CD4:CD8 ratio of 3.2:1 without any immunophenotypic aberrancies. Blasts were not identifiable in the analysis. The fluid cultures were negative for acid-fast bacilli, bacteria, and fungi.

The next day, a repeat TTE showed moderate to large-sized effusion. Due to the reaccumulation of the effusion, a decision was made to proceed with a pericardial window. Two drains were placed: a 28-French angled pericardial tube and a 16-French Blake tube in the anterior pericardial space. There were 300 cc of additional fluid collected. Flow cytometry of the pericardial fluid and pericardial biopsy confirmed the diagnosis of CLL/SLL with pericardial involvement, without evidence of large cell lymphoma transformation. The patient recovered well without subsequent complications and was discharged four days after the pericardial window.

He was discharged home and told to follow up with oncology for a repeat PET scan and discussions to initiate chemotherapy. The tentative plan was to begin fixed-duration venetoclax plus obinutuzumab given his FISH results from 2019 that showed peripheral blood positive for CD19, CD20, CD22, CD5, CD23, CD11c, CD200, and IgH somatic hypermutation with no pathogenic genetic alteration in TP53.

## Discussion

While malignancy is a common cause of large symptomatic pericardial effusions, CLL/SLL is hardly ever associated with cardiac tamponade [4]. Most patients with CLL/SLL are asymptomatic at the time of diagnosis and can live months or even years without requiring treatment. While CLL/SLL patients have higher odds of pericardial effusion compared to the average adult population [5], it is rarely seen as a consequence of disease progression. Upon reviewing the literature, it is clear that cardiac involvement related to CLL/SLL presents as heart failure, pericarditis, arrhythmia, or cardiac arrest, unlike our patient, who had cardiac tamponade secondary to a chronic effusion [6].

The clinical features of cardiac tamponade depend upon the onset and rate of fluid accumulation, with malignancy-related cases often resembling congestive heart failure (CHF). These can be differentiated by looking at B-type natriuretic peptide (BNP), response to diuretics, and TTE. Patients with CHF commonly have elevated BNP levels and usually respond to treatment with diuretics, neither of which was observed in our patient [7]. This case is a primary example of how cardiac tamponade can present in the absence of typical signs. On initial presentation, our patient had audible heart sounds and no jugular venous distension and was normotensive. His only positive findings were dyspnea, orthopnea, and pedal edema. Due to the slower rate of effusion in malignancies, the elastic fibrils adapt, maintaining normal hemodynamics with a higher volume-to-pressure ratio [8]. Meanwhile, rapid accumulation leads to swift hemodynamic compromise seen in typical cases of cardiac tamponade.

Management requires urgent fluid removal through percutaneous pericardiocentesis, increasing stroke volume and reducing intrapericardial and atrial pressures. This rapidly improves symptoms and hemodynamics, even if clinical or echocardiographic signs of cardiac tamponade persist. However, reaccumulation is common when effusion forms secondary to neoplasm. Therefore, patients with remaining pericardial effusion should be evaluated for invasive therapies, such as a pericardial window [9].

Pericardiocentesis with cytological and/or flow cytometry examination of the pericardial fluid should be performed in patients with hemorrhagic pericardial effusion. Cytological evaluation is especially critical in patients with underlying malignancy as in our patient. The sensitivity of cytology for the diagnosis of B-cell lymphoma is between 42.8% and 94.5% [10]. Positive cytology may be associated with poor outcomes in patients with neoplastic pericardial disease. Patients with positive cytology were also more likely to require repeat pericardiocentesis or surgical intervention.

Cardiac tamponade is an indication to begin chemotherapy for patients with CLL/SLL. Chemotherapy regimens must be individualized for this population and should exclude tyrosine kinase inhibitors such as ibrutinib given the increased risk for bleeding [11]. Furthermore, any patients already on anticoagulants or antiplatelet medications require careful monitoring for signs of effusion. Having a history of cardiac tamponade secondary to CLL/SLL underscores the need for individualized and closely managed treatment plans. Clinicians should navigate treatment decisions with a nuanced understanding of the patient's overall health, coexisting medical conditions, and the delicate balance between addressing CLL/SLL and managing the risk of bleeding complications, particularly in the context of pericardial involvement.

## Conclusions

Cardiac involvement in lymphoma is well-established; however, the occurrence of cardiac tamponade in CLL/SLL patients is exceptionally rare. Cardiac tamponade can be fatal, and clinicians should consider malignancy when evaluating patients with signs of pericardial effusions. An accurate and timely diagnosis of this rare manifestation of CLL/SLL can be lifesaving. It is important for clinicians treating patients with CLL/SLL to keep in mind that the cancer may affect multiple organs, including the heart. Early detection of CLL/SLL progression allows for proper initiation of therapy.
